# Supplementary material for: Experiences of HPV self-collection among Aboriginal and Torres Strait Islander women and people with a cervix
Source: PLoS One. 2026 Mar 9;21(3):e0326551. doi: 10.1371/journal.pone.0326551 (PMC12970916; doi:10.1371/journal.pone.0326551)
Supplement: S2 File — (PDF) [file pone.0326551.s002.pdf]

Supplementary material: Survey

Note. This survey was completed online using Qualtrics.

Start of Block: Information Sheet and Consent - All participants

Q0 **What is this survey about?**
We are inviting people to complete this 10-15 minute survey about preventing cervical cancer. You can do this survey if you:

- Are currently aged 24 to 74 years old
- Identify as a woman or have a cervix
- Live in Australia

This survey asks about your thoughts and feelings about cervical screening. It does not matter whether you have ever had a cervical screen or not.

<RESEARCHER DETAILS AND CONSENT FORM>

Q1.1 Please click the box below to help us prevent spam responses.

Q1.2 Would you like to take part in this survey?

- ○ I agree to take part in this survey (1)
- ○ I do not agree, and wish to exit this survey (0)

Skip To: End of Survey If Would you like to take part in this survey? = I do not agree, and wish to exit this survey

Q1.3 Where did you find out about this survey? (please be specific to help us prevent spam responses)

End of Block: Information Sheet and Consent - All participants

Start of Block: Eligibility - All participants (Part 1: About you)

Q80 **Part 1. About you**

Q2.1 Are you of Aboriginal or Torres Strait Islander origin?

- ○ Yes, Aboriginal (0)
- ○ Yes, Torres Strait Islander (2)
- ○ Yes, both Aboriginal and Torres Strait Islander (3)
- ○ Neither Aboriginal or Torres Strait Islander (4)

Q2.1 How old are you?

Skip To: End of Survey If Condition: How old are you? Is Greater Than 75. Skip To: End of Survey.

Skip To: End of Survey If Condition: How old are you? Is Less Than 24. Skip To: End of Survey.

Q2.5 How do you describe your gender?

Gender refers to current gender, which may be different to sex recorded at birth and may be different to what is on legal documents.

- ○ Woman or Female (1)
- ○ Man or Male (9)
- ○ Non-binary (10)
- ○ I use a different term (please specify) (11)
____
- ○ I prefer not to answer (99)

Q2.2 What was your sex recorded at birth?

- ○ Female (1)
- ○ Male (4)
- ○ Another term (please specify) (5) ____
- ○ I prefer not to answer (99)

Skip To: End of Survey If What was your sex recorded at birth? = Male

Q2.3 The cervix is the lower part of your uterus (or womb) which connects your uterus and vagina. If your sex recorded at birth was female, you were likely born with a womb and cervix.

The main reason for not having a cervix is if it has been removed by surgery. This is called a hysterectomy. You may still have a cervix if you have had a partial hysterectomy, where only the womb has been removed by surgery.

Do you have a cervix?

- ◯ Yes (1)
- ◯ No (0)
- ◯ Unsure (3)
- ◯ I prefer not to answer (99)

Skip To: End of Survey If The cervix is the lower part of your uterus (or womb) which connects your uterus and vagina. If y... = No

End of Block: Eligibility - All participants (Part 1: About you)

Start of Block: Demographic - All participants (Part 1: About you)

Q2.6 How do you describe your sexual orientation?

- ◯ Straight (Heterosexual) (1)
- ◯ Gay or lesbian (2)
- ◯ Bisexual (3)
- ◯ I use a different term (please specify) (4)
  ____
- ◯ I do not know (98)
- ◯ I prefer not to answer (99)

Q2.7 Were you born with a variation of sex characteristics (sometimes called ‘intersex’)?

- ◯ Yes (1)
- ◯ No (2)
- ◯ I do not know (3)
- ◯ I prefer not to answer (4)

Page Break

Q2.8 What is the highest level of education and training you have completed?

- ○ Did not go to school (1)
- ○ Primary school (2)
- ○ Some high school (i.e. Year 7 to Year 11, Form 1 to Form 5) (3)
- ○ Completed high school (i.e. Year 12, Form 6, HSC or equivalent, International Baccalaureate) (4)
- ○ TAFE, Trade Certificate or Diploma but did not complete Year 12 at secondary school (5)
- ○ TAFE or Trade Certificate or Diploma and also completed Year 12 at secondary school (6)
- ○ University, or some other Tertiary Institute degree, including post university (i.e. postgraduate diploma, master's degree, PhD) (7)
- ○ Other (please specify) (97) ____
- ○ I prefer not to answer (99)

Q2.9a What is your postcode?

____

Q2.9b What Australian state or territory do you live in?

- ○ Australian Capital Territory (1)
- ○ New South Wales (2)
- ○ Northern Territory (3)
- ○ Queensland (6)
- ○ South Australia (4)
- ○ Tasmania (5)
- ○ Victoria (8)
- ○ Western Australia (7)
- ○ I do not live in Australia (9)

Skip To: End of Survey If What Australian state or territory do you live in? = I do not live in Australia

Q2.10 What is your country of birth?

▼ Afghanistan (1) ... Zimbabwe (193)

Display This Question:

If List of Countries != Australia

Q2.11 If you were born overseas, how long have you been living in Australia?

- ◯ Less than a year (1)
- ◯ More than a year, please specify number of years: (2) ____
- ◯ Unsure/do not know (98)
- ◯ I prefer not to answer (99)

Q2.12 What is the main language you speak at home?

- ◯ English (1)
- ◯ Aboriginal language (13)
- ◯ Torres Strait Islander language (including Creole, Pidgin, Yumplatok) (14)
- ◯ Greek (2)
- ◯ Italian (3)
- ◯ Mandarin (4)
- ◯ Cantonese (5)
- ◯ Vietnamese (6)
- ◯ Arabic (7)
- ◯ Punjabi (8)
- ◯ Hindi (9)
- ◯ Tagalog (10)
- ◯ Other (please specify) (97) ____
- ◯ I prefer not to answer (99)

(Displayed only to Bots) Q1.4 Are you a human?

- ◯ Yes (1)

End of Block: Demographic - All participants (Part 1: About you)

Start of Block: Awareness of CSTs (Pt 2: Taking part in cervical screening)

Q92 **Part 2. Taking part in cervical screening**

Q3.1 Do you remember when your last cervical screening test was?

This might have been called a 'HPV test', 'Pap test' or 'Pap smear'.

- ◯ Within the last 18-months (on or after July 2022) (1)
- ◯ More than 18-months ago (Before July 2022) (2)
- ◯ I cannot remember when my last cervical screening test was (100)
- ◯ I have never had a cervical screening test (98)
- ◯ I have never heard of a cervical screening test (99)

Display This Question:

If Do you remember when your last cervical screening test was? This might have been called a 'HPV te... = Within the last 18-months (on or after July 2022)

Or Do you remember when your last cervical screening test was? This might have been called a 'HPV te... = More than 18-months ago (Before July 2022)

Q3.2 Where do you usually go for cervical screening?

- ◯ A doctor's clinic (1)
- ◯ A community health centre (2)
- ◯ A women's health centre (3)
- ◯ A family planning clinic (4)
- ◯ A sexual health clinic (5)
- ◯ An Aboriginal Medical Service or Aboriginal Community Controlled Health service (6)
- ◯ A gynaecologist (7)
- ◯ Other, please specify: (8) ____

End of Block: Awareness of CSTs (Pt 2: Taking part in cervical screening)

Start of Block: Awareness of CSTs- previously screened (Pt 2: Taking part in cervical screening)

Display This Question:

If Do you remember when your last cervical screening test was? This might have been called a 'HPV te... = Within the last 18-months (on or after July 2022)

Or Do you remember when your last cervical screening test was? This might have been called a 'HPV te... = More than 18-months ago (Before July 2022)

Q3.3 How often do you attend cervical screening/have a 'Pap test'?

- ◯ I screen around the time I am due (1)
- ◯ I know that sometimes I leave it for too long between screens (2)
- ◯ I have only had one screening test before (3)
- ◯ I prefer not to answer (5)

Display This Question:

If Do you remember when your last cervical screening test was? This might have been called a 'HPV te... = Within the last 18-months (on or after July 2022)

Or Do you remember when your last cervical screening test was? This might have been called a 'HPV te... = More than 18-months ago (Before July 2022)

Q3.4 What prompted you to get your last Cervical Screening Test or Pap Test?
You can select more than 1 option from the list below.

- ☐ I remembered I was due for a screening test (1)
- ☐ I went to the healthcare provider for another reason and they reminded me (2)
- ☐ The health worker told me I was due (100)
- ☐ A reminder letter, email, SMS (or similar) from my usual clinic (3)
- ☐ A reminder letter from the National Cancer Screening Register/Pap Test register (4)
- ☐ I wanted to follow-up after a previous abnormal cervical screen (5)
- ☐ I was worried about a symptom or health problem (6)
- ☐ I saw something in the media (television, radio, social media) (7)
- ☐ Other (please specify) (97) ____

- [ ] I cannot remember (98)
- [ ] I prefer not to answer (99)

End of Block: Awareness of CSTs- previously screened (Pt 2: Taking part in cervical screening)

Start of Block: Awareness of CSTs- never screened (Pt 2: Taking part in cervical screening)

Display This Question:

If Do you remember when your last cervical screening test was? This might have been called a 'HPV te... = I have never had a cervical screening test

Or Do you remember when your last cervical screening test was? This might have been called a 'HPV te... = I have never heard of a cervical screening test

Q3.5 Can you tell me your reasons for not doing a cervical screening test?
You can select more than 1 option from the list below.

- ▢ I do not have time (1)
- ▢ I forgot about it (2)
- ▢ I cannot get to the healthcare clinic (3)
- ▢ It is too expensive (4)
- ▢ There is not a healthcare provider near me who can do screening (5)
- ▢ I am scared of it (6)
- ▢ I am embarrassed/ashamed (7)
- ▢ I am worried it would be painful (8)
- ▢ I need more information about cervical screening before deciding (9)
- ▢ I do not want to have a healthcare provider touch me (10)
- ▢ I am worried I might be told I have cancer (11)
- ▢ I do not think I am at risk of cervical cancer/no family history (12)
- ▢ I have no symptoms (13)
- ▢ I am not currently sexually active (14)
- ▢ I have never been sexually active (15)
- ▢ I have had the Human Papillomavirus (HPV) vaccine (16)

- ☐ I did not know I should do screening (20)
- ☐ Other, please specify: (17) ____
- ☐ I do not know why (18)
- ☐ I prefer not to answer (19)

End of Block: Awareness of CSTs- never screened (Pt 2: Taking part in cervical screening)

Start of Block: Awareness of S.C- All (Pt 2: Taking part in cervical screening)

Q6.1 Have you ever heard of 'self-collection', 'self-sampling', or 'self-testing' as an option for cervical screening?

- ○ Yes (1)
- ○ No (0)
- ○ Unsure (98)
- ○ I prefer not to answer (99)

Display This Question:

If Have you ever heard of 'self-collection', 'self-sampling', or 'self-testing' as an option for cer... = Yes

Q6.2 How did you first hear about the self-collection testing option?
You can select more than 1 option from the list below.

- ▢ Family or friend (1)
- ▢ Healthcare provider told me about it (4)
- ▢ A letter or phone call from my healthcare clinic (100)
- ▢ Letter from the National Cancer Screening Register (5)
- ▢ Saw something in the media (television, radio, online news, print news) (6)
- ▢ Social media (99)
- ▢ Website (7)
- ▢ Campaign from an organisation such as Cancer Council, the Government or ACON (8)
- ▢ Other (please specify) (97) ____
- ▢ Unsure/cannot remember (98)

End of Block: Awareness of S.C- All (Pt 2: Taking part in cervical screening)

Start of Block: Information about cervical screening

Q0 **About cervical screening**

Please read the following information about cervical screening

At the end of 2017 Australia's National Cervical Screening Program changed from the Pap test, also known as a 'Pap smear', to a new, and better, cervical screening test that looks for human papillomavirus (HPV). HPV is a common virus that causes most cervical cancers. The newer cervical screening test for HPV is better at preventing cervical cancer than the Pap test, and also allows for samples to be collected in different ways.

Since July 2022, all people attending cervical screening can choose how they want their sample taken:

Option 1 is to take your own sample from the vagina using a soft swab which looks like a long cotton bud. This screening method is called 'self-collection'.

A healthcare provider can also help the person take their own sample, without a speculum, if the patient would like them to.

This is a self-collection soft swab.

Image from: The Australian Centre for the Prevention of Cervical Cancer.

Option 2 is to have a healthcare provider, like a doctor, nurse, or Aboriginal Health Practitioner, use the speculum. A speculum is a medical device that a healthcare professional uses to open the vagina so they can see the cervix and collect a cervical sample. This is the same way the Pap Test or Pap Smear was taken. In this survey we will call this a sample collected by a healthcare provider.

This is a speculum. It can be made from plastic or metal.

Image from: ShutterStock.

Both of these methods look for HPV and are available to women and people with a cervix for cervical screening.

End of Block: Information about cervical screening

Start of Block: Determine S.C Adoptor Status - Recently Screened (Pt. 3 Your cervical screening)

Display This Question:

If Do you remember when your last cervical screening test was? This might have been called a 'HPV te... = Within the last 18-months (on or after July 2022)

Q96 **Part 3. Your cervical screening options**

Display This Question:

If Do you remember when your last cervical screening test was? This might have been called a 'HPV te... = Within the last 18-months (on or after July 2022)

Q4.1 For your last cervical screening test, were you offered the choice between self-collection or having a sample collected by the healthcare provider using a speculum?

- ◯ Yes, I was offered a choice (1)
- ◯ No, I was not offered a choice (2)
- ◯ I cannot remember (3)
- ◯ I prefer not to answer (4)

Display This Question:

If Do you remember when your last cervical screening test was? This might have been called a 'HPV te... = Within the last 18-months (on or after July 2022)

Q4.2 How did you do your last cervical screening test?

- ○ I used self-collection in the clinic (1)
- ○ I used self-collection at home or somewhere else (2)
- ○ A healthcare provider helped me to collect my own sample (without a speculum) (3)
- ○ A healthcare provider collected the sample using a speculum (4)
- ○ I cannot remember (5)
- ○ I prefer not to answer (6)

End of Block: Determine S.C Adoptor Status - Recently Screened (Pt. 3 Your cervical screening)

Start of Block: Participants Recently Screened - SC Adopters (Pt 3. Your cervical screening)

Display This Question:

If How did you do your last cervical screening test? = I used self-collection in the clinic

Or How did you do your last cervical screening test? = I used self-collection at home or somewhere else

Q103 **Part 3. Your cervical screening options**

Display This Question:

If How did you do your last cervical screening test? = I used self-collection in the clinic

Or How did you do your last cervical screening test? = I used self-collection at home or somewhere else

Q4.3 Can you tell me your reasons for choosing to use self-collection?
You can select more than 1 option from the list below.

- ☐ It is less embarrassing (1)
- ☐ It is not as scary (4)
- ☐ It helps me feel in control of my body (5)
- ☐ It is less painful (6)
- ☐ I had a bad experience in the past when a sample was collected by a healthcare provider using a speculum (7)
- ☐ A friend/family member had a good experience with self-collection (8)
- ☐ It is just as accurate as a sample collected by a healthcare provider (9)
- ☐ It is more convenient (10)
- ☐ My healthcare provider suggested it as an option (11)
- ☐ The information I received at my screening appointment made it look like a good option (12)
- ☐ Other (please specify) (97) ____
- ☐ Unsure/cannot remember (98)
- ☐ I prefer not to answer (99)

End of Block: Participants Recently Screened - SC Adopters (Pt 3. Your cervical screening)

Start of Block: Resources Provided During Cervical Screen - All Recently Screened (Pt 3)

Display This Question:

If How did you do your last cervical screening test? = I used self-collection in the clinic

Or How did you do your last cervical screening test? = I used self-collection at home or somewhere else

Q4.4 Were you given the following information about self-collection during your last visit for cervical screening?

	Yes (1)	No (0)	Unsure/cannot remember (98)
That I might need to come back for another test with a healthcare provider if HPV was found (1)	○	○	○
That I could take the self-collection swab away and do it myself somewhere else (10)	○	○	○
That if I needed to have a follow up test, I might need to have a sample collected by a healthcare provider (11)	○	○	○
How I would get my test results (e.g SMS, phone call, letter) (12)	○	○	○
That self-collection was as accurate as a sample taken by a healthcare provider (13)	○	○	○
I had enough information to help me decide between self-collection and collection by a healthcare provider (14)	○	○	○
That I could ask for help with collecting the sample if I didn't feel comfortable (15)	○	○	○

Display This Question:

If How did you do your last cervical screening test? = I used self-collection in the clinic

Or How did you do your last cervical screening test? = I used self-collection at home or somewhere else

Q4.5 How much do you agree or disagree with the following statements?

Display This Choice:

If For your last cervical screening test, were you offered the choice between self-collection or hav... = Yes, I was offered a choice

Or For your last cervical screening test, were you offered the choice between self-collection or hav... = No, I was not offered a choice

Display This Choice:

If For your last cervical screening test, were you offered the choice between self-collection or hav... = Yes, I was offered a choice

Or For your last cervical screening test, were you offered the choice between self-collection or hav... = No, I was not offered a choice

Display This Choice:

If How did you do your last cervical screening test? = I used self-collection in the clinic

Or How did you do your last cervical screening test? = I used self-collection at home or somewhere else

Display This Choice:

If For your last cervical screening test, were you offered the choice between self-collection or hav... = Yes, I was offered a choice

Or For your last cervical screening test, were you offered the choice between self-collection or hav... = No, I was not offered a choice

Or For your last cervical screening test, were you offered the choice between self-collection or hav... = I cannot remember

Display This Choice:

If For your last cervical screening test, were you offered the choice between self-collection or hav... = Yes, I was offered a choice

Or For your last cervical screening test, were you offered the choice between self-collection or hav... = No, I was not offered a choice

Display This Choice:

If For your last cervical screening test, were you offered the choice between self-collection or hav... = Yes, I was offered a choice

Or For your last cervical screening test, were you offered the choice between self-collection or hav... = No, I was not offered a choice

Or For your last cervical screening test, were you offered the choice between self-collection or hav... = I cannot remember

	Agree (1)	Neither disagree nor agree (2)	Disagree (3)
Display This Choice:<br>*If For your last cervical screening test, were you offered the choice between self-collection or hav... = Yes, I was offered a choice*<br>*Or For your last cervical screening test, were you offered the choice between self-collection or hav... = No, I was not offered a choice*<br>I understood how to collect my own sample (1)	○	○	○
Display This Choice:<br>*If For your last cervical screening test, were you offered the choice between self-collection or hav... = Yes, I was offered a choice*<br>*Or For your last cervical screening test, were you offered the choice between self-collection or hav... = No, I was not offered a choice*<br>I was confident I could do the test correctly (9)	○	○	○
Display This Choice:<br>*If How did you do your last cervical screening test? = I used self-collection in the clinic*<br>*Or How did you do your last cervical screening test? = I used self-collection at home or somewhere else*<br>The information I was given was easy to understand (15)	○	○	○
Display This Choice:<br>*If For your last cervical screening test, were you offered the choice between self-collection or hav... = Yes, I was offered a choice*<br>*Or For your last cervical screening test, were you offered the choice between self-collection or hav... = No, I was not offered a choice*<br>*Or For your last cervical screening test, were you offered the choice between self-collection or hav... = I cannot remember*<br>I was confident the test was accurate (10)	○	○	○

Display This Choice:<br>*If For your last cervical screening test, were you offered the choice between self-collection or hav... = Yes, I was offered a choice*<br>*Or For your last cervical screening test, were you offered the choice between self-collection or hav... = No, I was not offered a choice*<br>It was easy for me to collect the sample (11)	○	○	○
Display This Choice:<br>*If For your last cervical screening test, were you offered the choice between self-collection or hav... = Yes, I was offered a choice*<br>*Or For your last cervical screening test, were you offered the choice between self-collection or hav... = No, I was not offered a choice*<br>*Or For your last cervical screening test, were you offered the choice between self-collection or hav... = I cannot remember*<br>I was worried about having to return for a follow up test (12)	○	○	○

Page Break

Display This Question:

If How did you do your last cervical screening test? = I used self-collection in the clinic

Or How did you do your last cervical screening test? = I used self-collection at home or somewhere else

Q4.6 If self-collection was **not** an option, how likely is it that you would have screened?

- ◯ I would not have had cervical screening without self-collection (1)
- ◯ I might have had cervical screening without self-collection (2)
- ◯ I would have had cervical screening anyway (3)

End of Block: Resources Provided During Cervical Screen - All Recently Screened (Pt 3)

Start of Block: Participant recently screened with clinician-collection (Pt 3)

Display This Question:

If How did you do your last cervical screening test? = A healthcare provider collected the sample using a speculum

Q104 **Part 3. Your cervical screening options**

Display This Question:

If How did you do your last cervical screening test? = A healthcare provider collected the sample using a speculum

Q4.7 Can you tell me your reasons for choosing to have a sample collected by the healthcare provider using a speculum?
You can select more than 1 option from the list below.

- ▢ I was not told about the option to use self-collection (1)
- ▢ I have always had it done by a healthcare provider (2)
- ▢ I wanted my healthcare provider to have a look (3)
- ▢ My healthcare provider told me they did not offer self-collection (4)
- ▢ I am not eligible for self-collection (5)
- ▢ I needed more information about self-collection (6)
- ▢ I did not think I would take the self-collected sample properly (7)
- ▢ I did not think self-collection was accurate (8)
- ▢ I thought I would need to return for another test anyway if I used self-collection (9)
- ▢ Other (please specify) (10) ____
- ▢ I do not know why (11)
- ▢ I prefer not to answer (12)

End of Block: Participant recently screened with clinician-collection (Pt 3)

Start of Block: Participants recently screened- SC Adopters (clinician-assisted) (Pt 3)

Display This Question:

If How did you do your last cervical screening test? = A healthcare provider helped me to collect my own sample (without a speculum)

Q105 **Part 3. Your cervical screening options**

Display This Question:

If How did you do your last cervical screening test? = A healthcare provider helped me to collect my own sample (without a speculum)

Q4.8 Can you tell me your reasons for choosing to have your sample collected by a healthcare provider using a self-collection swab?
You can select more than 1 option from the list below

- ▢ It's less embarrassing (1)
- ▢ I thought that the healthcare provider could collect a better sample than I could (2)
- ▢ I want my healthcare provider to have a look, even without a speculum, and make sure that everything is ok (3)
- ▢ I cannot reach, or it is not comfortable for me to reach (4)
- ▢ I do not like to touch myself there (5)
- ▢ Other (please specify) (6) ____
- ▢ Unsure/ do not remember (7)
- ▢ I prefer not to answer (8)

End of Block: Participants recently screened- SC Adopters (clinician-assisted) (Pt 3)

Start of Block: Participants previously screened (Pt 3. Your cervical screening)

Display This Question:

If Do you remember when your last cervical screening test was? This might have been called a 'HPV te... = Within the last 18-months (on or after July 2022)

Or Do you remember when your last cervical screening test was? This might have been called a 'HPV te... = More than 18-months ago (Before July 2022)

Q4.9 How would you prefer to do your next cervical screen?

- ○ Collected by a healthcare provider using a speculum (1)
- ○ Collected by a healthcare provider using a vaginal swab (but no speculum) (2)
- ○ Self-collection (3)
- ○ I would be okay with either (4)
- ○ I am not planning to screen (5)
- ○ I do not know (6)
- ○ I prefer not to answer (7)

End of Block: Participants previously screened (Pt 3. Your cervical screening)

Start of Block: Never screened (Pt 3. Your cervical screening)

Display This Question:

If Do you remember when your last cervical screening test was? This might have been called a 'HPV te... = I cannot remember when my last cervical screening test was

Or Do you remember when your last cervical screening test was? This might have been called a 'HPV te... = I have never had a cervical screening test

Or Do you remember when your last cervical screening test was? This might have been called a 'HPV te... = I have never heard of a cervical screening test

Q106 **Part 3. Your cervical screening options**

Display This Question:

If Do you remember when your last cervical screening test was? This might have been called a 'HPV te... = I cannot remember when my last cervical screening test was

Or Do you remember when your last cervical screening test was? This might have been called a 'HPV te... = I have never had a cervical screening test

Or Do you remember when your last cervical screening test was? This might have been called a 'HPV te... = I have never heard of a cervical screening test

Q4.10 Do you think you are more likely to take part in cervical screening because self-collection is available?

- ○ Yes, I am more likely to have a cervical screening test (1)
- ○ No, I still think I would not have a cervical screening test (2)
- ○ It does not affect my decision to have a cervical screening test or not (3)
- ○ I am not sure (4)
- ○ I prefer not to answer (5)

End of Block: Never screened (Pt 3. Your cervical screening)

Start of Block: All participants (Pt 3. Your cervical screening)

Q4.11 How important are the following things to you when it comes to cervical screening?

	Very important (1)	Important (2)	Not important (3)	Not sure or not applicable (4)
Having the choice over how I can do cervical screening (1)	○	○	○	○
Being able to talk with my healthcare provider about cervical screening (2)	○	○	○	○
Having simple and clear information on cervical screening (3)	○	○	○	○
Having my healthcare provider explain self-collection to me (4)	○	○	○	○
Having the healthcare provider available if I needed help doing self-collection (5)	○	○	○	○
Feeling safe and comfortable within the clinic (11)	○	○	○	○
Feeling safe and comfortable with the healthcare provider (6)	○	○	○	○
Having the choice of healthcare provider (for example, a doctor, nurse or health worker) (7)	○	○	○	○
Having a female healthcare provider (8)	○	○	○	○
Having flexible options for how I can access cervical screening (for example, in the clinic or at home) (9)	○	○	○	○

4.12 Who would you prefer to talk with about cervical screening?

- My usual doctor (male or female) (1)
- A female doctor (2)
- Nurse (3)
- Midwife (4)
- Health worker (5)
- Aboriginal Health Worker/Practitioner (6)
- Other (please specify): (9) ____
- I do not mind (7)
- I prefer not to answer (8)

End of Block: All participants (Pt 3. Your cervical screening)

Start of Block: All participants (Pt 4. Future screening preferences)

Q5 **Part 4. Taking part in cervical screening in the future**

There are just a few more questions left about self-collection. Even if you would prefer a healthcare provider to collect the sample using a speculum for your cervical screening test, we would like to know more about how you would prefer to pick up a self-collection swab, collect the sample, and return the swab.

Q5.1 Where would you most prefer to do self-collection (collect the sample yourself)?

- At my healthcare provider's clinic during an appointment (1)
- At the place I picked up the swab from (e.g. doctor's office, a pathology pick-up point) (2)
- At home (3)
- Somewhere else, please specify: (4) ____
- I have no preference (5)

Q5.2 If you were to do the test at home, how would you prefer to return the self-collection swab for it to be tested?

- ○ By mail with a prepaid envelope (1)
- ○ Drop it off at my healthcare provider's clinic (2)
- ○ Drop it off at a pathology collection centre (e.g. where you go to get a blood test) (3)
- ○ Somewhere else, please specify: (5) ____
- ○ I have no preference (4)

Page Break

Q5.3 How likely would you be to take part in cervical screening on time compared to now if the following options for self-collection were available?

	More likely (1)	Same (4)	Less likely (5)	This does not apply to me (6)
Receiving the self-collection swab in the post after a telehealth appointment (phone or video call with your healthcare provider) (1)	○	○	○	○
Collecting the self-collection swab from the doctor's office or pathology pick-up point after a telehealth appointment (2)	○	○	○	○
Collecting the self-collection swab from my doctor's office/medical service without an appointment (3)	○	○	○	○
Collecting the self-collection swab from a health worker during a routine health assessment (13)	○	○	○	○
Picking up a self-collection swab from my Aboriginal medical service (14)	○	○	○	○
Picking up a self-collection swab from the chemist/pharmacy (7)	○	○	○	○
Ordering a self-collection swab online/over the phone when I am due for cervical screening (4)	○	○	○	○
Automatically receiving a self-collection swab in the mail when I am due for screening (5)	○	○	○	○
Picking up a self-collection swab at community events (6)	○	○	○	○
Picking up a self-collection swab when I attend breast screening (when you are aged 50-74) (8)	○	○	○	○
Getting a self-collection swab when I receive my bowel screening kit (when you are aged 50-74) (11)	○	○	○	○

Q5.4 How would you most prefer to get the swab for self-collection?

○ Receiving the self-collection swab in the post after a telehealth appointment (phone or video call with your healthcare provider) (1)

○ Collecting the self-collection swab from the doctor's office or pathology pick-up point after a telehealth appointment (2)

- ○ Collecting the self-collection swab from my doctor's office/medical service without an appointment (3)
- ○ Collecting the self-collection swab from a health worker during a routine health assessment (13)
- ○ Picking up a self-collection swab from my Aboriginal medical service (14)
- ○ Picking up a self-collection swab from the chemist/pharmacy (7)
- ○ Ordering a self-collection swab online/over the phone when I am due for cervical screening (4)
- ○ Automatically receiving a self-collection swab in the mail when I am due for screening (5)
- ○ Picking up a self-collection swab at community events (6)
- ○ Picking up a self-collection swab when I attend breast screening (when you are aged 50-74) (8)
- ○ Getting a self-collection swab when I receive my bowel screening kit (when you are aged 50-74) (11)

Q5.5 Why is this your most preferred way of getting the swab for self-collection? You can select more than 1 option from the list below:

- ☐ It is closer to home (10)
- ☐ I can get it outside of work hours (11)
- ☐ I can bring my family or children with me (12)
- ☐ It is more convenient (1)
- ☐ It is less embarrassing (2)
- ☐ It is less expensive (3)
- ☐ I prefer to talk to a healthcare provider about cervical screening (4)
- ☐ I have a trusted healthcare provider (5)
- ☐ I do not have a trusted healthcare provider (6)
- ☐ I am not sure (7)
- ☐ Other, please specify: (8) ____
- ☐ I prefer not to answer (9)

End of Block: All participants (Pt 4. Future screening preferences)

Start of Block: All participants (Pt 5. End of survey)

Q107 **Part 5. End of survey**

Q6.1 Would you like to enter the prize draw for a $100 prepaid Visa gift card? You will be asked to provide your email address.

- Yes (1)
- No (2)

Q6.2 Would you like to receive a copy of the survey results? You will be asked to provide your email address.

- Yes (1)
- No (2)

Display This Question:

If Would you like to enter the prize draw for a $100 prepaid Visa gift card? You will be asked to pr... = Yes

Or Would you like to receive a copy of the survey results? You will be asked to provide your email a... = Yes

Q6.4 Please enter your email address so the researchers can contact you.

Your email address will only be used to let you know if you have won a voucher or send you a copy of the survey results. It will not be used to identify you or your answers.

____

Q6.3 Where did you find out about this survey? (We are asking this a second time to help identify spam responses)

____

End of Block: All participants (Pt 5. End of survey)
