# Supplementary material for: Experiences of HPV self-collection among Aboriginal and Torres Strait Islander women and people with a cervix
Source: PLoS One. 2026 Mar 9;21(3):e0326551. doi: 10.1371/journal.pone.0326551 (PMC12970916; doi:10.1371/journal.pone.0326551)
Supplement: Attachment — Submitted filename: Review.docx [file pone.0326551.s004.docx]

Review: Experiences of HPV self-collection among Aboriginal and Torres Strait Islander women and people with a cervix

Introduction: Maybe a small excerpt on HPV infection prevalence amongst First Nations People in Australia will be valuable in providing context, could be added along with the cervical cancer burden bit.

Page 3 - Line 19-20 – please add “People” after Islander.

Page 4 - The last paragraph of the introduction uses terminology “Aboriginal and/or Torres Strait Islander women”. I would suggest using uniform terminology throughout manuscript, i.e use of Aboriginal and Torres Strait Islander People or Aboriginal and/or Torres Strait Islander people.

Page 4 – line 17 ,18,19 – do not seem to be an adequate ending for the paragraph. I would suggest finishing the paragraph at HPV Self collection (i.e. since July 2022). And add the following details into the methodology section.

Methodology – I would recommend adding more details about the Advisory group and Indigenous governance of the project. Even though the positionality statement specifies some details, it is important for the non-Indigenous readers to understand the value and significance of having Indigenous Governance.

I would suggest moving the ethical approval section after positionality instead at the end of the methodology section.

Results- I think it would be important to recognise the number of participants who had been assessed by a HCP before, to see how many preferred to still go with the HCP and how many preferred a self-collection. For some women it may be a first time experience, and in that case the motives for a self-collection are different. Whereas the women who had previous history of a HCP collecting a sample and then opted for a self-collection could also provide insights to the perception of patients regarding the mannerisms portrayed by the HCP and how comfortable or culturally safe did they feel with the HCP in the health care services.

Was any qualitative data also collected as a part of this study? To explore the patient experiences? I can understand that it could beyond the scope of this paper, but probably citing it within the result or methods section would be helpful for readers.
