# Supplementary material for: Experiences of HPV self-collection among Aboriginal and Torres Strait Islander women and people with a cervix
Source: PLoS One. 2026 Mar 9;21(3):e0326551. doi: 10.1371/journal.pone.0326551 (PMC12970916; doi:10.1371/journal.pone.0326551)
Supplement: Attachment — Submitted filename: PONE-D-25-29330_AuthorResponse.docx [file pone.0326551.s005.docx]

**Author Response Manuscript ID:** PONE-D-25-29330

**Title:** Experiences of HPV self-collection among Aboriginal and Torres Strait Islander women

and people with a cervix

| Reviewer Comment | Author Response | Manuscript Location |
| --- | --- | --- |
| Reviewer 1: | | |
| *Comments to the Author | Thank you for your feedback. |  |
| **General Comments** Suggest that you include the questionnaire as an appendix as that would provide a suitable resource for other Indigenous groups to consider using some or all of the questions to assess self-sampling in their populations | Noted, this has been uploaded as an appendix file rather than supplemental file. |  |
| **Introduction**  The background information provided important details on changes to the cervical cancer screening guidelines in Australia over the past 10 years. The rationale for evaluating self-sampling in the Aboriginal and Torres Strait Islander was reasonably supported but whether this change will overcome barriers to participation by these people was not as clearly established. | This has been amended to include the broader context in which self-collection is offered: Universal access to self-collection provides an opportunity to address screening inequities among Aboriginal and Torres Strait Islander women and people with a cervix. However, it must not be implemented in isolation. Without addressing systemic issues, such as racism, access to culturally safe care, and the ongoing impacts of colonisation, inequities in cervical screening participation are likely to persist. | Page 4, line 20 |
| The cited literature was relevant and generally very recent; however, there was a lack of information about HPV vaccinations uptake in this population. Please include additional information as that is likely most relevant in the 25–35-year-olds mentioned on page 15 beginning on line 14 regarding positive oncogenic HPV screens. | We have incorporated literature regarding HPV vaccination to strengthen the evidence base and contextualise its role alongside cervical screening strategies | Page 3 line 12 and line 21 |
| M**aterials and Methods**  Page 5 line 2 – an electronic version of the survey will not be accessible to people without access to computers and the internet. How would this potentially bias the results in general but specifically this population who tend to live in more remote areas? | We have added this as a study limitation and suggested that additional research utilising separate methods targeting these groups would be needed | Page 22, line 25 |
| Page 6 line 5 – how were responses kept separate from participants enrolling in the draw for a voucher? Was only one voucher offered for all respondents to the survey? | We have provided additional detail about how this was managed. To maintain confidentiality, one study member (CJ) managed a separate database for the voucher draw, ensuring that survey data remained independent from participant contact details; 12 vouchers were available exclusively for Aboriginal and Torres Strait Islander participants. Random selection function was used in excel for the prize draw. The prize draw was completed within 1 month from the final dataset being downloaded, and participant contact details were deleted following this | Page 7, line 2 |
| **Table 1**  Many of the reported counts for variable categories do not sum up to the column totals. Please include a category for missing data so that it is clear to the reader if questions were not answered. Data suppression is fine but likely not needed for missing information. | Missing data has been added to Table 1. | Page 9, line 13 |
| How is remoteness defined? | Postcode was used to classify the participant’s place of residence by geographic remoteness using Australian Statistical Geography Standard Remoteness Structure derived from Accessibility/Remoteness Index of Australia Plus (ARIA+). This is described however we then grouped into subcategories so this has been described. We have now described this in the methods and Table 1. | Page 8, line 20 and Table 1 |
| **Table 2**  **M**any of the reported counts for variable categories do not sum up to the column totals. Please include a category for missing data so that it is clear to the reader if questions were not answered. | This has been added | Table 2, Page 12 |
| Are the categories for the question “What prompted you to get a CST “ mutually exclusive? It does not seem so. Please include information about the question in the table, for example add (select all that apply). | This has been added | Table 2, Page 12 |
| Add a row total to this table as results for individual questions are reported for the combined groups in the Screening Practices section. | This has been added | Table 2, Page 12 |
| Could report some differences between the two groups, such as “Around when due for screening“ and “I was worried about a symptom or health problem.” The reasons for these differences could then be explored in the Discussion. | Additional detail has been provided and raised in the discussion. | Page 11, Lines 8-15 |
| Table 3 – add a column with the sample size for each question rather than including it with the question As with Tables 1 and 2, many of the reported counts for variable categories do not sum up to the sample sizes.  Please include a category for missing data so that it is clear to the reader if questions were not answered. | Table 3 from the original paper has been split into Table 3: Collection method of recently screened participants and Table 4: Reasons for collection method, with information about missing respondents provided to improve clarity. | Table 3, Page 13 and Table 4, Page 15-16 |
| Are the categories for the questions “Reasons for choosing HCP assisted self-collection (i.e. no speculum “ and “Reasons for choosing self-collection” mutually exclusive? It does not seem so. Please include information about the question in the table, for example add (select all that apply). The footnote for this table is helpful but adding information to the question makes this easier to understand too. | This has been included in the table to improve clarity | Table 4, Page 15-16 |
| Why is the sample size 89 for this question “Reasons for choosing self-collection” when it seems it should be 116? Is this the number who answered this question out of the 116 respondents? | Survey logic meant that only participants who collected their own sample were asked this series of questions – therefore only 89 were offered this question. This is clarified in the text. | Page 14, line 2 |
| It would be more informative to look at the responses to this question separately for the groups who were or were not offered a choice for screening: “Reasons for choosing a clinician-collected sample (i.e. with a speculum).” The reasons might be different and that could inform who to target with educational materials in the future. | Table 4 now looks at reasons for selected method by the offer of the choice of collection method. The most frequent response for those who chose clinician collection by choice was “always having it done by a healthcare provider” or “wanting them to have a look”, | Page 15-16 |
| Page 13 lines 3 to 13: the counts and percentages presented here do not tally with the numbers in table 3, unless these are the results only from those who answered these questions, not whether they did self-collection. For example, Screened using self-collection^ should be 116 in total not 89. Please include more information on these results (e.g., 89 out of possible 116 answered this question ….) as they are difficult to interpret and understand as they are written now. | See above comment re Table 3 and Table 4. We have split the information into two tables and moved the paragraphs to correspond to each table which should improve clarity. The 89 referenced in the text refers to the 89 participants who collected their own sample either at home or in the clinic, whereas the 118 refers to those who used any method of self-collection and includes those who had a healthcare provider assist them without a speculum (n=29). This has also been amended to improve flow and clarity. | Table 3, Page 13 |
| Figure 2 – the figure legend should indicate the results are given as percentages. It should also indicate multiple responses are possible and why the results do not sum to 100%. The graph currently shows the total is 100% but it is not for the data presented. Including a missing or not answered category would improve the interpretation of these results. The figure is also quite blurry.  Results from this Table 2 can be shown better using multiple bar charts, side by side. See this web page for an example: <https://communities.sas.com/t5/Graphics-Programming/Multiple-Bar-charts-side-by-side/td-p/277586> | The layout of the figure has been modified and split into Figure 2 and 3 to improve comprehension.  We have chosen horizonal bar charts given the large volume of category text. | Page 29 |
| **Discussion/Conclusions**  Page 16 Lines 11-19- Are there challenges with access to primary care in this population due to remote locations or fewer options? Or was your sample more likely from an urban setting (see comment about study limitation below)? | Additional information has been provided | Page 21, Line 7 |
| Page 17 line 15 “… in a major (city)”? | This has been amended |  |
| Strengths and Limitations section – only mentions strengths. A major limitation is self-report information was collected from a non-probabilistic, convenience sample. The data cannot be verified using actual examination data. Another limitation is the use of an electronic version of the survey that would likely exclude people who don’t have access to a computer and the internet. Another potential limitation is differential recall bias – the people recently screened will likely have more accurate recall of their appointment compared to someone who might have been screened several years ago. | These limitations have been described | Page 22, line 15 |
| The Discussion does not describe how the results of this study could improve participation by people who have never screened or are not up to date in their screening? Does the National Campaign that started in 2024 target these groups? Or their healthcare providers? Please expand so that the knowledge gained from this survey can improve cervical cancer screening in this population, and possibly the more general screen-eligible population in Australia. | Additional information has been provided | Page 22, line 3 |
| **Reviewer 2:** |  |  |
| Introduction: Maybe a small excerpt on HPV infection prevalence amongst First Nations People in Australia will be valuable in providing context, could be added along with the cervical cancer burden bit. | We have provided data on the rates of oncogenic HPV detected among Aboriginal and Torres Strait Islander peoples as part of cervical screening to provide context. | Page 3, line 21 |
| Page 3 - Line 19-20 – please add “People” after Islander. | This has been added |  |
| Page 4 - The last paragraph of the introduction uses terminology “Aboriginal and/or Torres Strait Islander women”. I would suggest using uniform terminology throughout manuscript, i.e use of Aboriginal and Torres Strait Islander People or Aboriginal and/or Torres Strait Islander people. | This has been amended for consistency. |  |
| Page 4 – line 17 ,18,19 – do not seem to be an adequate ending for the paragraph. I would suggest finishing the paragraph at HPV Self collection (i.e. since July 2022). And add the following details into the methodology section | The research questions have been moved into Data Analysis | Page 8 line 10 |
| Methodology – I would recommend adding more details about the Advisory group and Indigenous governance of the project. Even though the positionality statement specifies some details, it is important for the non-Indigenous readers to understand the value and significance of having Indigenous Governance. | Thank you for this suggestion. While we had strong Governance processes in place, we had not detailed it in this manuscript. This has been added as a section | Page 5, line 15. |
| I would suggest moving the ethical approval section after positionality instead at the end of the methodology section. | This has been moved. | Page 5, line 22 |
| Results- I think it would be important to recognise the number of participants who had been assessed by a HCP before, to see how many preferred to still go with the HCP and how many preferred a self-collection. For some women it may be a first time experience, and in that case the motives for a self-collection are different. Whereas the women who had previous history of a HCP collecting a sample and then opted for a self-collection could also provide insights to the perception of patients regarding the mannerisms portrayed by the HCP and how comfortable or culturally safe did they feel with the HCP in the health care services. | This has been added to the results sections (see page 13, lines 7-16) and the discussion (page 18 lined 9-16) |  |
| Was any qualitative data also collected as a part of this study? To explore the patient experiences? I can understand that it could beyond the scope of this paper, but probably citing it within the result or methods section would be helpful for readers | This has been added as a study limitation. | Page 23, line 16 |

Thank you for your consideration of this manuscript.
