# Supplementary material for: Experiences of HPV self-collection among Aboriginal and Torres Strait Islander women and people with a cervix
Source: PLoS One. 2026 Mar 9;21(3):e0326551. doi: 10.1371/journal.pone.0326551 (PMC12970916; doi:10.1371/journal.pone.0326551)
Supplement: Attachment — Submitted filename: Response To Reviewers.docx [file pone.0326551.s006.docx]

PONE-D-25-29330R1: Experiences of HPV self-collection among Aboriginal and Torres Strait Islander women and people with a cervix

PLOS One

Thank you for your consideration of our manuscript. We submit a revised version incorporating the below comments:

| Journal Requirements: |  |
| --- | --- |
| 1. If the reviewer comments include a recommendation to cite specific previously published works, please review and evaluate these publications to determine whether they are relevant and should be cited. There is no requirement to cite these works unless the editor has indicated otherwise. | Noted. |
| 2. Please review your reference list to ensure that it is complete and correct. If you have cited papers that have been retracted, please include the rationale for doing so in the manuscript text, or remove these references and replace them with relevant current references. Any changes to the reference list should be mentioned in the rebuttal letter that accompanies your revised manuscript. If you need to cite a retracted article, indicate the article’s retracted status in the References list and also include a citation and full reference for the retraction notice. | We have carefully reviewed our reference list and confirm there are no retracted papers included. |
| Additional Editor Comments: |  |
| Thank you for submitting the revised version of your manuscript PONE-D-25-29330R1 entitled "Experiences of HPV self-collection among Aboriginal and Torres Strait Islander women and people with a cervix" to PLOS ONE. I have now completed my evaluation of your revisions and am pleased to inform you that the changes satisfactorily address the concerns raised by the reviewers. On this basis, I am happy to provisionally accept your manuscript. | Thank you for your feedback. We look forward to publishing this manuscript and appreciate your consideration. |
| Reviewer #1: |  |
| Thank you for carefully addressing my previous comments and suggestions. I found the revised manuscript to be easier to follow and the additional content improved my understanding of the research study. | Thank you for your feedback. |
| I have two additional comments based on reading the revised manuscript: 1. The last paragraph on page 13 of track-changed version of the manuscript seems to require clarification in order to understand what is being presented. This is the sentence that seems to need some additional explanation: “The majority of these participants (61%, n=81) were not offered a choice of collection method, however 38% (n=50) participants were offered a choice and elected to have a clinician-collected sample, and an additional two couldn’t remember.” | We have provided additional detail to explain this sentence (located line 3, page 13): The majority of participants *who screened using clinician-collection* (61%, n=81) were not offered a choice of collection method. However 50 participants were offered a choice and elected to have a clinician-collected sample, and an additional two couldn’t remember. |
| 2. On page 20, this sentence would benefit from the addition of the word 'group': 21 Interestingly, the majority of this GROUP had previously screened and ...” | This has been included (line 26, page 18). |
